# Identifying Highly Conserved and Highly Differentiated Gene Ontology Categories in Human Populations

**DOI:** 10.1371/journal.pone.0027871

**Published:** 2011-11-30

**Authors:** Yongshuai Jiang, Ruijie Zhang, Peng Sun, Guoping Tang, Xuehong Zhang, Xing Wang, Xiaodan Guo, Qiuyu Wang, Xia Li

**Affiliations:** College of Bioinformatics Science and Technology, Harbin Medical University, Harbin, China; Wayne State University, United States of America

## Abstract

Detecting and interpreting certain system-level characteristics associated with human population genetic differences is a challenge for human geneticists. In this study, we conducted a population genetic study using the HapMap genotype data to identify certain special Gene Ontology (GO) categories associated with high/low genetic difference among 11 Hapmap populations. Initially, the genetic differences in each gene region among these populations were measured using allele frequency, linkage disequilibrium (LD) pattern, and transferability of tagSNPs. The associations between each GO term and these genetic differences were then identified. The results showed that cellular process, catalytic activity, binding, and some of their sub-terms were associated with high levels of genetic difference, and genes involved in these functional categories displayed, on average, high genetic diversity among different populations. By contrast, multicellular organismal processes, molecular transducer activity, and some of their sub-terms were associated with low levels of genetic difference. In particular, the neurological system process under the multicellular organismal process category had low levels of genetic difference; the neurological function also showed high evolutionary conservation between species in some previous studies. These results may provide a new insight into the understanding of human evolutionary history at the system-level.

## Introduction

With the development of high throughput single nucleotide polymorphism (SNP) genotyping technology, the identification of millions of SNPs facilitated population genetics studies and medical genetics research, such as designing and analyzing genome-wide association studies based on HapMap genotype data [1], [2], identifying recombination hot spots [3], searching for signals of evolutionary selection [4], and analyzing demographic history [5]. A total of 40.8% of human SNPs distribute in gene regions and 59.2% SNPs are in the intergene regions. The SNP density in the gene region is slightly higher than in the intergene region [6]. Over the past few years, studies have compared the SNPs in certain gene regions, such as the vitamin D receptor (VDR) gene region [7], drug related gene regions [8], and the enzyme glucokinase (GCK) gene region [9], and found patterns of genetic variation among human populations. Although these studies provided an important contribution to understanding the human genome, they only considered one or a few gene regions. A group of genes often work together to affect a given biological function or process; therefore, understanding an event at the organismal level requires analysis of many genes, rather than the analysis of individual genes. Annotation databases, such as GO [10], [11], [12], [13] and KEGG [14], [15], [16], [17], provide important resources for system-level studies. Recently, some studies have focused on certain general system-level characteristics of species evolution [18], [19]. They have successfully identified biological pathways that have high or low evolutionary conservation by comparing homologous proteins. A study of human-rodent orthologs indicated that genes in GO function category with neurological associations exhibited high evolutionary conservation, and had lower K_A_/K_S_ ratios [18]. Another study indicated that GO categories associated with regulatory processes (such as signal transducers, transcription factors, and receptors) and responses to the environment (such as defense response, immune response, and response to stimulus) were evolving rapidly [19]. Although some special gene functional categories associated with long-term species evolution have been studied in great detail, there have been few studies of gene functional categories associated with the short-term human population differentiation. In fact, human populations live in variable environments, and many layers of demographic and evolutionary events, such as migrations, population expansions, colonizations, genetic drift, selection, recombination and mutation, have shaped human genetic variation [20].

Are there some functional gene sets associated with high/low genetic differences among human populations? Here, we conducted a population genetic study to find GO categories associated with genetic differences among different populations. First, for each autosome gene region among 11 HapMap populations, we measured the differences in SNPs in each gene region using selected indicators, such as the allele frequency, LD pattern, and transferability of tag SNPs, which were usually used for comparing samples from different populations [21], [22], [23], [24], [25], [26] and reflected population genetic characteristics. We then tested the associations between GO functional categories and population genetic differences to identify GO categories associated with high or low levels of genetic difference among different populations.

## Materials and Methods

### Data

#### HapMap genotype data

In this study, we used public data from the HapMap project. The international HapMap project, launched in 2002, is an international effort to document the common SNPs in the human genome [27], [28], [29], [30]. Currently, the HapMap includes 11 sample populations: African ancestry in Southwest USA (ASW), Utah residents with Northern and Western European ancestry from the CEPH collection (CEU), Han Chinese in Beijing, China (CHB), Chinese in Metropolitan Denver, Colorado (CHD), Gujarati Indians in Houston, Texas (GIH), Japanese in Tokyo, Japan (JPT), Luhya in Webuye, Kenya (LWK), Mexican ancestry in Los Angeles, California (MEX), Maasai in Kinyawa, Kenya (MKK), Toscans in Italy (TSI), and Yoruba in Ibadan, Nigeria (YRI). We selected 1,002 unrelated individuals and 1,063,592 autosomal SNPs in all 11 HapMap populations. 987,019 SNPs passed quality control (QC) criteria: Hardy-Weinberg equilibrium (HWE) p>0.001 in an individual population, call frequency >0.75, and minor allele frequency (MAF) >0.01 (Table 1).

**Table 1:** Summary of HapMap data.

HapMap populations	ASW	CEU	CHB	CHD	GIH	JPT	LWK	MEX	MKK	TSI	YRI	total
Number of HapMap samples	83	174	86	85	88	89	90	77	171	88	176	1207
Number of Unrelated individuals	49	116	86	85	88	89	90	50	143	88	118	1002
SNPs in all 11 populations	1,063,592											
SNPs passed QC	987,019											

#### Human genome data

A total of 30,770 entries for autosomal gene information were extracted from the “seq-gene” file downloaded from the NCBI ftp website. All records include chromosome, chr_start, chr_stop, feature_id (NCBI gene ID), “feature_type” of “gene” and “group_label” of “reference”. Genes that had more than one chromosome location were removed in our study. The average size of these 30,770 genes was 38,353 bp.

#### GO data

The GO project is a collaborative effort to develop and use ontologies to support biologically meaningful annotation of genes and their products [31]. It develops three ontologies of defined biological descriptors (GO terms) representing gene product properties: biological process (BP), describing a broad biological objective; molecular function (MF), describing the elemental activities of a gene product at the molecular level; and cellular component (CC), describing the location of the gene product [32]. Each ontology is structured as a directed acyclic graph. In this study, each GO category that was considered as a functional gene set was used to identify the association with genetic differences among the 11 HapMap populations.

The “term” file (the definitions of each node or term) and the “graph_path” file (the parent-child relationships for each node) were downloaded from the Gene Ontology website. To associate the GO categories with gene IDs, the file “gene2go” was downloaded from the NCBI ftp. These files were downloaded on April 29, 2011. There were some entries which do not have support evidences, such as entries with Evidence codes: “NAS” (non-traceable author statement) and “ND” (no biological data available were removed). These entries were removed from “gene2go”. Finally, the BP, MF, and CC Ontologies had associations with 12,990, 14,046, and 15,413 genes, respectively.

### Calculating genetic differences among 11 HapMap populations based on allele frequencies

Human population originated from the same ancestors, and the differences of allele frequency between different populations were the result of population differentiation. The allele frequency as a population genetic characteristic was usually used for comparing samples from different populations [21], [22], [23], [24], [25], [26]. Because of linkage equilibrium, there were some correlations between alleles of SNPs in close proximity on a chromosome [33], [34], and the average population differences of these adjacent SNPs may represent the characteristics of the entire region. Therefore, we measured the average differences of allele frequency for each gene region between pair-wise HapMap populations. For each gene region, we defined the difference of allele frequency 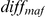 as follows:
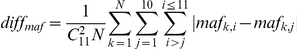
Where 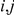 are HapMap populations (1:ASW, 2:CEU, 3:CHB, 4:CHD, 5:GIH, 6:JPT, 7: LWK, 8:MEX, 9:MKK, 10:TSI, 11:YRI). 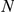 is the number of SNPs in a gene region. 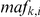 is the frequency of the 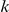
th SNP in population 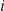, 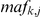 is the frequency of the 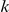
th SNP in population 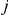. A larger 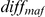 indicates a larger difference in allele frequency in the gene region among 11 HapMap populations; a smaller 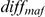 indicates a smaller difference.

### Calculating genetic differences among 11 HapMap populations based on LD patterns

For each gene region, four indicators of the LD pattern were calculated. (1) LD coefficient r^2^ (r^2^). We calculated pairwise LD coefficients (r^2^) between all pairwise SNPs (less than 500 kb). (2) Average block size (block_size). For each gene region, a Four Gamete Test (FGT) [35] was used to identify the haplotype block structure, and the average size of the blocks within the gene region was calculated. (3) Average SNP density of blocks (SNP_dens). (4) Average haplotype diversity (hap_div). For each block in each gene region, haplotype diversity [24] was computed as 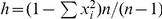, where 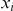 was the frequency of a given haplotype and 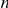 was the number of samples, and average haplotype diversity was defined as the average value of haplotype diversity in the block regions. In this study, haploview v4.1 [36] was used to identify haplotype block and to estimate haplotype frequency (haplotype frequency >1%) using the expectation-Maximization (EM) algorithm. The differences of the four indicators among 11 HapMap populations (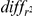, 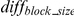, 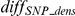 and 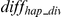) were calculated in the same way as 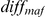.

### Calculating genetic differences among 11 HapMap populations based on transferability of tagSNPs

There were three indicators of the transferability of tagSNPs (a representative single nucleotide polymorphism (SNP) in a region of the genome with high linkage disequilibrium). (1) Tag Percent (tag_perc). For each gene region, an aggressive tagging strategy by the TAGGER panel in haploview was used to identify tagSNPs (r^2^ threshold is 0.8). The tag percent was defined as the number of tagSNPs divided by the total number of SNPs in a gene region. (2) Captured percent (Cap_perc). For example, for the ASW population, if an ASW SNP exhibited a pairwise r^2^>0.8 with at least one tagSNP selected from the CEU population, then the SNP was defined as a captured SNP by the CEU panel in the ASW population [24]. The captured percent was defined as the number of captured SNPs divided by the total number of SNPs in ASW population. (3) Average maximum r^2^ (max_ r^2^). For each gene region, the average maximum r^2^ was defined as the average value of the maximum r^2^ between tagSNPs in one HapMap population and SNPs captured by these tagSNPs in another population. Captured percent and Average maximum r^2^ were used to evaluate the efficiency of tagSNPs in one population to capture SNPs in another population. The differences of the three indicators among 11 HapMap populations (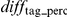, 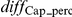 and 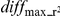) were calculated in the same way as 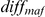. The above eight indicators were calculated for genes containing at least two SNPs.

### Identifying Gene Ontology categories associated with genetic differences among 11 HapMap populations

The genetic differences of a GO category were reflected by combining the differences of all genes in that GO category. Some previous studies demonstrated that genes assigned to the same GO category are more closely related in terms of some aspect of their biology than random sets of genes [37], [38]. To identify GO categories associated with genetic differences among the 11 HapMap populations, firstly, we annotated the genes listed in gene2go by the GO terms associated with the genes and by the complete hierarchy of parent terms; only GO categories containing at least ten genes were analyzed. Secondly, for each GO category, we assigned the same weight to genes belonging to the GO category and calculated genetic difference scores for each of the eight indicators separately. In this way, we combined the genetic differences of genes in the corresponding GO category. Genetic difference scores of the GO category for each of the eight indicators were defined as follows:
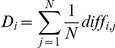
Where 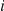 is indicator name (1: maf, 2: r^2^, 3: block_size, 4: SNP_dens, 5: hap_div, 6: tag_perc, 7: cap_perc, 8: max_ r^2^), 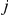 is the 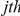 gene in a GO category, 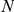 is the gene number in the GO category and 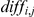 is the 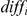 for gene 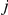. 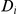 was used to measure the GO category difference among 11 HapMap populations. Finally, for each GO category, we randomly picked the same number of genes from one of three ontologies (BP, MF or CC) and recalculated 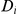. The entire procedure was repeated 10,000 times to obtain the random background distribution of 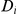. After testing for normality with the Kolmogorov-Smirnov test, we found that the background distribution of 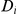 was approximately normal. The probability of the left side was used to identify GO categories associated with low levels of genetic difference among 11 HapMap populations, and the probability of the right side was used to identify GO categories associated with high levels of genetic difference. The significance level 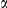 was 0.01. To obtain robust conclusions, we imposed a seriously restricted condition: for a GO category, only when eight indicators were all significant in the left/right side, was the GO category associated with low/high level of genetic difference among 11 HapMap populations.

For example, GO:0016192 (vesicle-mediated transport, includes 720 genes) is a sub-term of biological process (12,990 genes), the 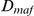 was 0.136. We randomly picked 720 genes from the 12,990 genes and recalculated 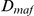 10,000 times to construct the random background distribution. The random background values of 10,000 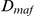 approximately obeyed normal distribution (Figure S1), and p-value (right side) was 5.336E-07. The other seven p-values were 1.370E-06 for r^2^, 1.290E-06 for block_size, 5.624E-03 for Snp_dens, 6.690E-03 for hap_div, 1.500E-06 for tag_perc, 5.505E-10 for Cap_perc, and 2.588E-06 for max_ r^2^. The eight p-values were all less than 0.01; therefore, we believed that the GO: 0016192 was associated with high levels of genetic difference among the 11 HapMap populations.

## Results

We chose to analyze 4,875 GO categories containing at least ten genes: BP, 3,546 categories; MF, 831 categories; and CC, 498 categories. In total, 67 GO terms were associated with differences among the 11 HapMap populations (all eight p-values <0.01). 50 GO categories (BP, 16 GO terms; MF, 15 GO terms; and CC, 19 GO terms) were associated with high levels of genetic difference among the 11 HapMap populations and 17 GO categories (BP, 7 GO terms; MF, 6 GO terms; and CC, 4 GO terms) were associated with low levels of genetic difference.

### GO terms associated with high levels of genetic difference among 11 hapmap populations

**Figure 1:**
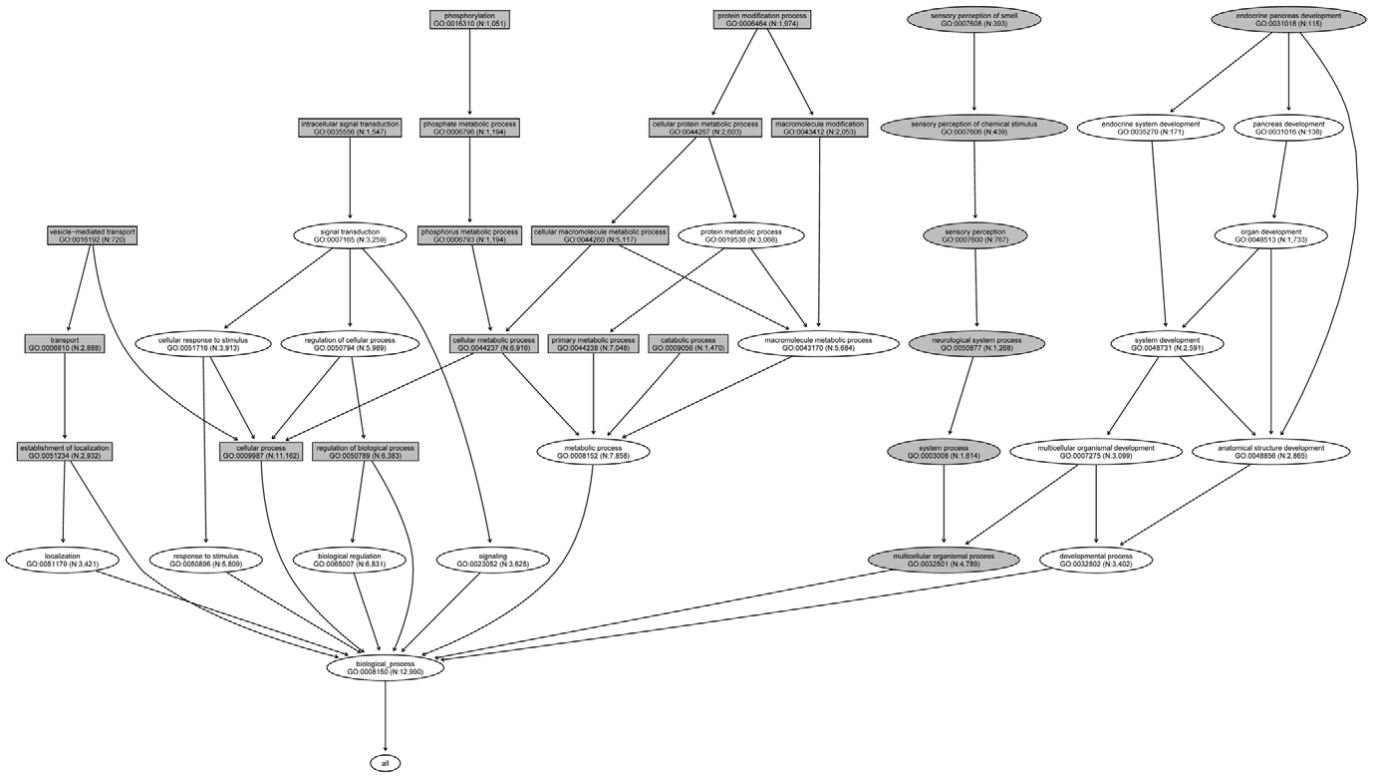
Biological process GO terms associated with high and low levels of genetic difference (“gray ellipse” nodes represent the low difference GO terms, and “gray rectangle” nodes represent the high difference GO terms). N represents the number of genes in a GO term.

For biological processes, there were 16 GO terms that were associated with high levels of difference among the 11 HapMap populations (Table S1). The 16 GO terms had lower right side probability p-values (p<0.01) for all eight indicators. To find relationship among the GO terms, a GO Slim was created to generate a highly aggregated report of GO categories associated with the high levels of population genetic difference (Figure 1). “GO Slim” is a simplified version of GO that combines and removes fine grained terms in GO [39]. The parent-child relationships in a GO Slim could provide a global view for significant GO terms. The parent would be a broader GO term, and the child would be a more specific term. We found that most of the GO terms (10 GO terms) were encompassed in metabolic process (GO:0008152, Figure 1) and cellular process (GO:0009987, Figure 1). Catabolic process (GO:0009056), cellular metabolic process (GO:0044237), and primary metabolic process (GO:0044238) were the main metabolic process categories associated with high levels of genetic difference.

**Figure 2:**
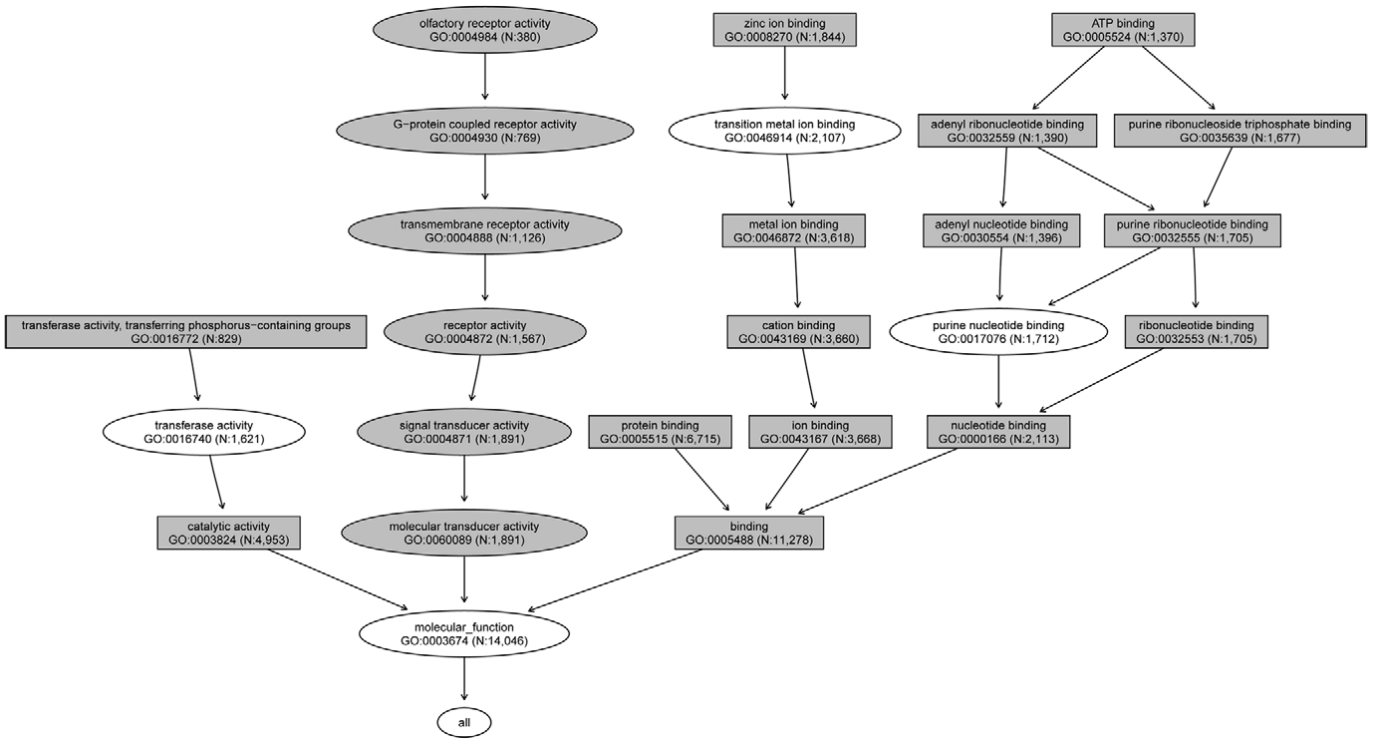
Molecular function GO terms associated with high and low levels of genetic difference (“gray ellipse” nodes represent the low difference GO terms, and “gray rectangle” nodes represent the high difference GO terms). N represents the number of genes in a GO term.

For molecular function, there were 15 GO terms that were associated with high levels of genetic difference among the 11 HapMap populations (Table S1). All GO terms were encompassed in two GO categories: catalytic activity (GO:0003824) and binding (GO:0005488) (Figure 2). The former contained 4,953 genes, and all eight p-values were less than 0.01 (0.000 for maf, 7.772E-16 for r^2^, 0.000 for block_size, 1.563E-03 for SNP_dens, 2.666E-08 for hap_div, 0.000 for tag_perc, 0.000 for cap_perc, and 9.959E-14 for max_ r^2^). The latter contained 11,278 genes, and the eight p-values were 0.000 for maf, 0.000 for r^2^, 0.000 for block_size, 2.062E-10 for SNP_dens, 1.110E-15 for hap_div, 0.000 for tag_perc, 0.000 for cap_perc, and 0.000 for max_ r^2^. This category had 51 first-level subnodes, three of which were associated with high levels of genetic difference. They were GO:0000166: nucleotide binding, GO:0005515: protein binding, and GO:0043167: ion binding.

**Figure 3:**
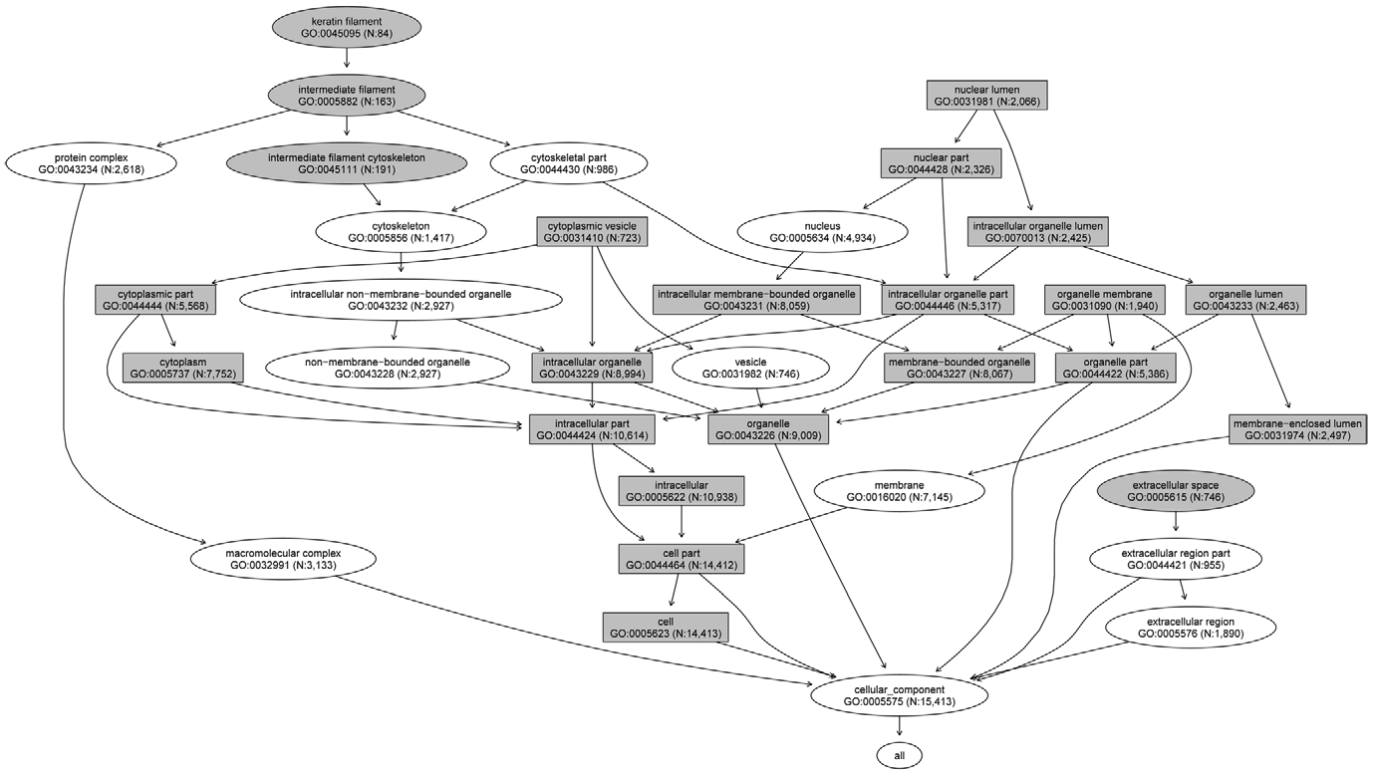
Cellular component GO terms associated with high and low levels of genetic difference (“gray ellipse” nodes represent the low difference GO terms, and “gray rectangle” nodes represent the high difference GO terms). N represents the number of genes in a GO term.

For cellular component, there were 19 GO terms that were associated with high levels of genetic difference (Table S1). Most of the GO terms were encompassed in two GO categories: cell (GO:0005623) and organelle (GO:0043226) (Figure 3). The former contained 14,413 genes and the latter contained 9,009 genes. All eight p-values for the two categories showed strong association with high levels of genetic difference among 11 HapMap populations.

### GO terms associated with low levels of genetic difference among 11 HapMap populations

For biological process, there were 7 GO terms that were associated with low levels of genetic difference (Table S2). The seven GO terms had lower left side probability p-values (p<0.01) for all eight indicators. Most of the GO terms were encompassed in multicellular organismal process (Figure 1, GO:0032501), an important sub-category of which was neurological system process (GO:0050877). A series of GO categories (sensory perception (GO:0007600), sensory perception of chemical stimulus (GO:0007606), and sensory perception of smell (GO:0007608), encompassed in the neurological system process category, showed strong association with low levels of difference among the 11 HapMap populations.

For molecular function, there were 6 GO terms that were associated with low levels of genetic difference among the 11 HapMap populations (Table S2). An interesting result was that all the GO terms were encompassed in molecular transducer activity (GO:0060089, Figure 2). The categories encompassed in the GO category were signal transducer activity (GO:0004871), receptor activity (GO:0004872), transmembrane receptor activity (GO:0004888), G-protein coupled receptor activity (GO:0004930), and olfactory receptor activity (GO:0004984).

For cellular component, there were 4 GO terms that were associated with low levels of genetic difference among the 11 HapMap populations (Table S2). They were intermediate filament (GO:0005882), extracellular space (GO:0005615), keratin filament (GO:0045095), and intermediate filament cytoskeleton (GO:0045111) (Figure 3).

To analyze the effects of the gene number of the GO categories on our results, the Pearson's correlation coefficients between p-values and the gene numbers of the GO categories were calculated. Table S3 shows that all eight Pearson's correlation coefficients were lower between p-values and gene numbers of GO categories. The maximum correlation coefficient is 0.170 (SNP_dens). This indicated that the number of genes had no effect on our results.

We also analyzed the correlation between indicators. The Pearson's correlation coefficients in an 8 by 8 matrix from all eight indicators were computed (Table S4). Table S4 shows that most of the correlation coefficients were between 0 and 0.5, and all the correlation coefficients were less than 0.8. The minimum cut-off value of 0.8 for correlation coefficients is usually used to identify the correlations between indicators [40], [41]. These indicators did not show high correlations, and they reflected different population genetic characteristics.

## Discussion

In this study, we assessed the genetic differences among populations for each autosome gene region and identified GO categories associated with these genetic differences. First, for each gene region, the differences in SNPs were measured using the allele frequency, the LD pattern, and transferability of tag SNPs. However, genes are not independent of each other; a group of genes often acts together to perform a specific biological task. Thus, each GO category that was considered as a functional gene set was used to identify the association with population genetic differences. Finally, we identified special functional groups that were associated with population genetic differences.

The GO categories associated with high genetic differences among the 11 HapMap populations mainly belonged to six root nodes: metabolic process (BP, Figure 1), cellular process (BP, Figure 1), catalytic activity (MF, Figure 2), binding (MF, Figure 2), cell (CC, Figure 3), and organelle (CC, Figure 3). Although metabolic processes have showed evolutionary conservation between species in some previous studies [19], we found that some sub-processes, such as catabolic process, cellular metabolic process, and primary metabolic process, were associated with high levels of genetic difference among different human populations. This might be because these functional categories had been subjected to different selection pressures in the different environments in which ancient human populations resided, such as climate, diet, and pathogens [20], [42]. The different conservation patterns between and among species will help geneticists understand the evolution of species and the population differentiation within species. In a previous study, some “binding” categories, such as “protein binding” [43], exhibited rapid evolution among species. The present study showed that the category “protein binding” was associated with high levels of genetic difference among human populations.

The GO categories associated with lower levels of difference among the 11 HapMap populations mainly belonged to two root nodes: the multicellular organismal process category (BP, Figure 1) and the molecular transducer activity category (MF, Figure 2). The neurological system process category (BP), under the biological process node multicellular organismal process category (BP), was associated with lower levels of genetic difference among different populations. The neurological function category was also associated high levels of evolutionary conservation between species in some previous studies, and neurologically relevant genes had lower K_A_/K_S_ ratios [18]. For the signal transducer activity category, the conservation of signal transduction pathways had been previously observed [44], [45]. Although the sensory perception of smell category (BP) and the olfactory receptor activity category (MF) belonged to different ontologies (BF and MF), they were both associated with human olfactory function. A study of genes for insect olfaction demonstrated high levels of functional conservation across 250 million years of evolution [46]. In this study, we also found similar results in human: these categories showed lower levels of difference among the 11 HapMap populations.

In summary, these GO categories that are associated with high or low levels of genetic difference will help geneticists explore differentiation among and between human populations, and may provide useful clues in the understanding of human evolutionary history from system-level.

In addition, our results have practical implications for disease association studies, such as genome wide association (GWA) studies. Association analysis is a powerful method for identifying genes involved in complex disorders. Recently, GWA studies have been successful in identifying susceptibility genes for several complex disorders [47], [48], [49], [50]. However, the population differences in allele frequencies and LD structure may affect the power of associations analysis; association signals for markers may appear at different positions because of different populations' LD structures [51]. For gene regions associated with lower levels of genetic difference, if a SNP is identified to be associated with a disease, the SNP will probably be a risk marker in another population. However, for regions associated with higher levels of difference, we must consider the effect of population structure, and some statistical method should be used to decrease the effect [52]. In this study, we only investigated the gene regions; however, their adjacent regions (such as 10 kbp, 100 kbp) should be considered in association analysis. Furthermore, association analysis also focuses on searching for the association signal of pathways [38], [53].

In this study, we investigated the average differences among 11 HapMap populations. In the future, we will investigate the differences between pair-wise populations, respectively, and we hope that future research on genes and their adjacent regions will be of benefit to GWA studies.

### Web Resources

The URLs for the data presented herein are as follows:

1. HapMap. Available:http://hapmap.ncbi.nlm.nih.gov/. Accessed 2011 April 29.
2. GO database. Available:http://www.geneontology.org. Accessed 2011 April 29.
3. NCBI. Available:http://www.ncbi.nlm.nih.gov. Accessed 2011 April 29.
4. NCBI seq_gene download site. Available:ftp://ftp.ncbi.nih.gov/genomes/H_sapiens/mapview/. Accessed 2011 April 29.
5. NCBI gene2go file download site. Available:ftp://ftp.ncbi.nih.gov/gene/DATA. Accessed 2011 April 29.

## Supporting Information

Figure S1
**Distribution of 10,000** 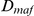 **values.**
(TIF)

Table S1 **GO terms associated with high genetic differences among 11 HapMap populations.**
(DOC)

Table S2 **GO terms associated with low genetic differences among 11 HapMap populations.**
(DOC)

Table S3 **Pearson's correlation coefficients between p-values and gene numbers of GO categories.**
(DOC)

Table S4 **Correlation coefficients matrix for eight indicators.**
(DOC)
