# Supplementary figures and images for: Identifying Highly Conserved and Highly Differentiated Gene Ontology Categories in Human Populations
Source: PLoS One. 2011 Nov 30;6(11):e27871. doi: 10.1371/journal.pone.0027871 (PMC3227580; doi:10.1371/journal.pone.0027871)

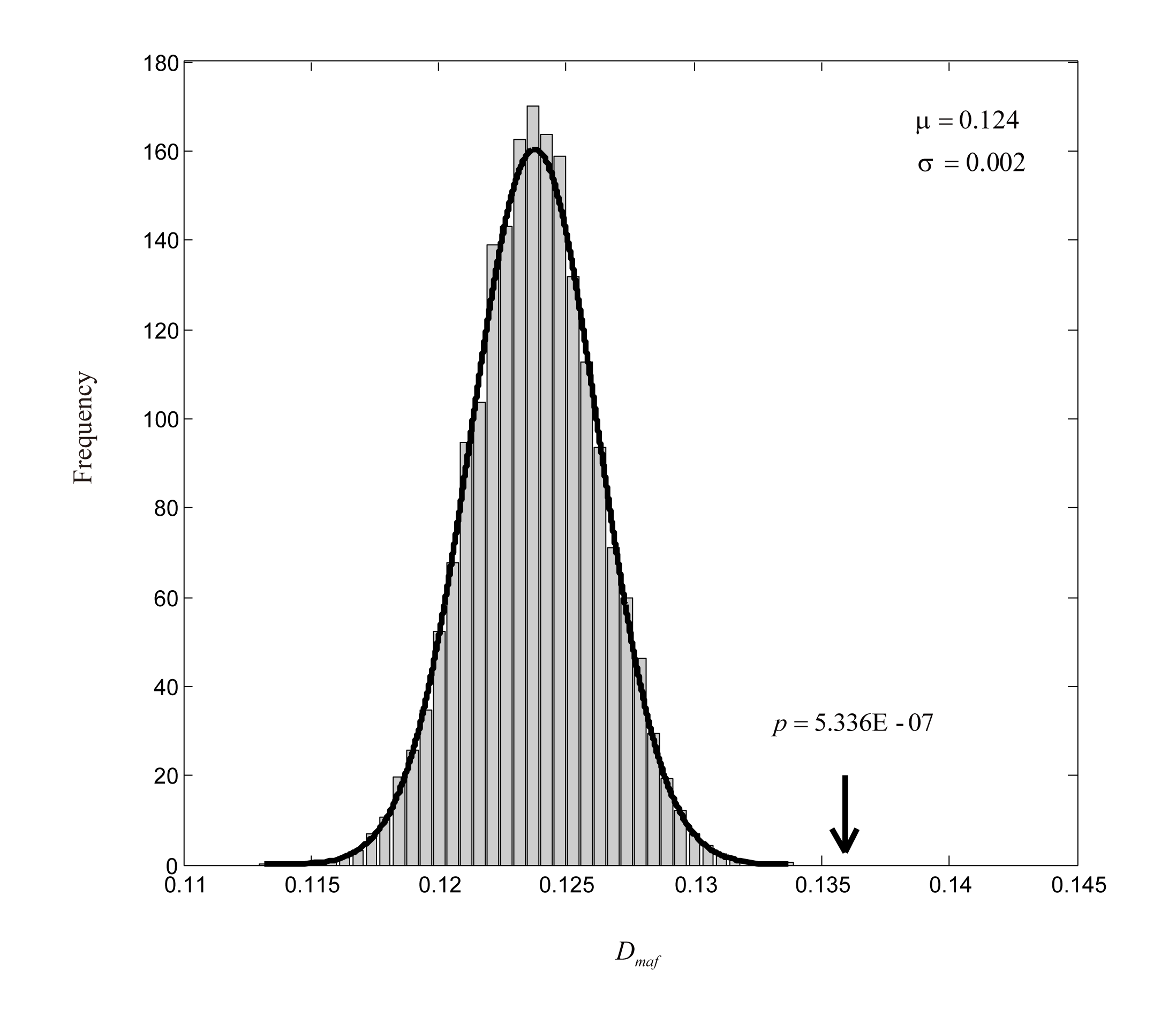

Supplement: Figure S1 — Distribution of 10,000 values. (TIF) [file pone.0027871.s001.tif]
