# Supplementary material for: Identifying Highly Conserved and Highly Differentiated Gene Ontology Categories in Human Populations
Source: PLoS One. 2011 Nov 30;6(11):e27871. doi: 10.1371/journal.pone.0027871 (PMC3227580; doi:10.1371/journal.pone.0027871)
Supplement: Table S1 — GO terms associated with high genetic differences among 11 HapMap populations. (DOC) [file pone.0027871.s002.doc]

**Supplementary table 1.** GO terms associated with high genetic differences among 11 HapMap populations.

| GO ID | Term name | Gene  number | Right side p-values | | | | | | | |
| --- | --- | --- | --- | --- | --- | --- | --- | --- | --- | --- |
|  |  |  | Maf | r^2^ | block_size | Snp_dens | hap_div | tag_perc | Cap_perc | max_r^2^ |
| Biological process (16 terms) | |  |  |  |  |  |  |  |  |  |
| GO:0016192 | vesicle-mediated transport | 720 | 5.336E-07 | 1.370E-06 | 1.290E-06 | 5.624E-03 | 6.690E-03 | 1.500E-06 | 5.505E-10 | 2.588E-06 |
| GO:0035556 | intracellular signal transduction | 1,547 | 2.188E-07 | 4.743E-03 | 3.387E-07 | 1.315E-03 | 7.722E-04 | 1.684E-07 | 6.295E-10 | 2.451E-05 |
| GO:0006464 | protein modification process | 1,974 | 2.319E-12 | 1.606E-10 | 0.000E+00 | 3.169E-04 | 7.687E-07 | 2.070E-08 | 2.220E-16 | 9.047E-08 |
| GO:0006793 | phosphorus metabolic process | 1,194 | 1.324E-06 | 6.777E-04 | 2.209E-14 | 4.245E-03 | 4.625E-03 | 7.090E-07 | 4.387E-11 | 1.608E-04 |
| GO:0006796 | phosphate metabolic process | 1,194 | 1.324E-06 | 6.777E-04 | 2.209E-14 | 4.245E-03 | 4.625E-03 | 7.090E-07 | 4.387E-11 | 1.608E-04 |
| GO:0006810 | Transport | 2,888 | 1.059E-11 | 8.589E-06 | 6.737E-04 | 6.406E-04 | 3.344E-03 | 1.112E-11 | 2.263E-13 | 1.476E-09 |
| GO:0009056 | catabolic process | 1,470 | 9.186E-05 | 3.585E-05 | 3.662E-10 | 5.480E-03 | 3.892E-04 | 6.602E-03 | 2.877E-04 | 4.374E-03 |
| GO:0009987 | cellular process | 11,162 | 0.000E+00 | 0.000E+00 | 0.000E+00 | 1.299E-12 | 0.000E+00 | 0.000E+00 | 0.000E+00 | 0.000E+00 |
| GO:0016310 | Phosphorylation | 1,051 | 3.018E-05 | 6.337E-03 | 5.132E-11 | 4.765E-03 | 4.779E-03 | 6.780E-06 | 1.298E-08 | 1.078E-03 |
| GO:0043412 | macromolecule modification | 2,053 | 1.061E-12 | 2.975E-11 | 0.000E+00 | 4.122E-04 | 1.596E-07 | 9.837E-09 | 0.000E+00 | 2.659E-08 |
| GO:0044237 | cellular metabolic process | 6,916 | 1.079E-12 | 0.000E+00 | 3.331E-15 | 1.229E-03 | 1.110E-16 | 2.051E-06 | 1.420E-08 | 3.115E-11 |
| GO:0044238 | primary metabolic process | 7,048 | 1.687E-10 | 0.000E+00 | 7.216E-15 | 3.747E-03 | 2.129E-13 | 2.721E-06 | 8.513E-09 | 3.895E-09 |
| GO:0044260 | cellular macromolecule metabolic process | 5,117 | 8.384E-07 | 2.665E-15 | 0.000E+00 | 1.068E-03 | 1.854E-14 | 8.648E-03 | 8.459E-05 | 1.023E-06 |
| GO:0044267 | cellular protein metabolic process | 2,603 | 1.125E-08 | 6.082E-12 | 0.000E+00 | 4.149E-03 | 9.001E-09 | 1.035E-04 | 8.994E-10 | 4.207E-06 |
| GO:0050789 | regulation of biological process | 6,383 | 6.142E-04 | 9.319E-03 | 4.443E-03 | 3.848E-05 | 5.089E-03 | 7.042E-05 | 4.677E-04 | 6.874E-05 |
| GO:0051234 | Establishment of localization | 2,932 | 3.539E-12 | 7.894E-06 | 2.869E-04 | 5.929E-04 | 2.305E-03 | 5.444E-12 | 9.270E-14 | 1.659E-09 |
| Molecular function (15 terms) | |  |  |  |  |  |  |  |  |  |
| GO:0000166 | nucleotide binding | 2,113 | 1.110E-16 | 4.834E-09 | 0.000E+00 | 5.507E-03 | 1.053E-05 | 5.910E-09 | 0.000E+00 | 7.668E-08 |
| GO:0005515 | protein binding | 6,715 | 1.110E-16 | 3.821E-11 | 0.000E+00 | 1.285E-05 | 6.858E-11 | 1.117E-08 | 1.310E-14 | 1.456E-10 |
| GO:0003824 | Catalytic activity | 4,953 | 0.000E+00 | 7.772E-16 | 0.000E+00 | 1.563E-03 | 2.666E-08 | 0.000E+00 | 0.000E+00 | 9.959E-14 |
| GO:0005488 | Binding | 11,278 | 0.000E+00 | 0.000E+00 | 0.000E+00 | 2.062E-10 | 1.110E-15 | 0.000E+00 | 0.000E+00 | 0.000E+00 |
| GO:0046872 | metal ion binding | 3,618 | 0.000E+00 | 3.066E-09 | 7.067E-05 | 3.014E-03 | 8.444E-06 | 4.685E-14 | 2.109E-15 | 2.200E-11 |
| GO:0005524 | ATP binding | 1,370 | 0.000E+00 | 4.851E-08 | 0.000E+00 | 3.486E-04 | 3.852E-04 | 8.436E-11 | 0.000E+00 | 1.120E-07 |
| GO:0008270 | zinc ion binding | 1,844 | 3.807E-08 | 1.556E-09 | 1.438E-04 | 2.959E-03 | 4.141E-08 | 7.996E-04 | 2.262E-03 | 2.868E-06 |
| GO:0016772 | transferase activity, transferring phosphorus-containing groups | 829 | 4.581E-11 | 3.047E-04 | 0.000E+00 | 4.761E-03 | 3.420E-03 | 9.553E-07 | 4.297E-14 | 7.552E-04 |
| GO:0030554 | adenyl nucleotide binding | 1,396 | 0.000E+00 | 1.294E-07 | 0.000E+00 | 6.013E-04 | 3.958E-04 | 1.522E-10 | 0.000E+00 | 1.980E-07 |
| GO:0032553 | ribonucleotide binding | 1,705 | 9.881E-15 | 2.318E-07 | 0.000E+00 | 9.185E-03 | 6.776E-04 | 3.686E-09 | 0.000E+00 | 7.222E-07 |
| GO:0032555 | purine ribonucleotide binding | 1,705 | 9.881E-15 | 2.318E-07 | 0.000E+00 | 9.185E-03 | 6.776E-04 | 3.686E-09 | 0.000E+00 | 7.222E-07 |
| GO:0032559 | adenyl ribonucleotide binding | 1,390 | 0.000E+00 | 9.994E-08 | 0.000E+00 | 4.961E-04 | 3.197E-04 | 8.678E-11 | 0.000E+00 | 1.593E-07 |
| GO:0035639 | purine ribonucleoside triphosphate binding | 1,677 | 8.549E-15 | 1.219E-07 | 0.000E+00 | 6.614E-03 | 6.151E-04 | 6.568E-09 | 0.000E+00 | 6.568E-07 |
| GO:0043167 | ion binding | 3,668 | 0.000E+00 | 1.461E-09 | 1.148E-04 | 3.179E-03 | 6.376E-06 | 1.355E-14 | 3.331E-16 | 2.013E-11 |
| GO:0043169 | cation binding | 3,660 | 0.000E+00 | 1.517E-09 | 9.500E-05 | 3.539E-03 | 7.315E-06 | 1.721E-14 | 4.441E-16 | 2.129E-11 |
| Cellular component (19 terms) | |  |  |  |  |  |  |  |  |  |
| GO:0005622 | intracellular | 10,938 | 0.000E+00 | 0.000E+00 | 0.000E+00 | 2.165E-07 | 0.000E+00 | 0.000E+00 | 0.000E+00 | 0.000E+00 |
| GO:0005623 | cell | 14,413 | 5.551E-15 | 1.110E-16 | 0.000E+00 | 2.812E-07 | 1.986E-07 | 1.243E-10 | 0.000E+00 | 1.598E-09 |
| GO:0005737 | cytoplasm | 7,752 | 0.000E+00 | 0.000E+00 | 0.000E+00 | 3.165E-09 | 0.000E+00 | 0.000E+00 | 0.000E+00 | 0.000E+00 |
| GO:0031090 | organelle membrane | 1,940 | 1.331E-08 | 2.722E-09 | 5.964E-04 | 4.507E-03 | 3.100E-06 | 4.632E-06 | 2.479E-09 | 9.216E-06 |
| GO:0031410 | cytoplasmic vesicle | 723 | 3.874E-05 | 3.167E-04 | 8.012E-03 | 4.221E-03 | 4.237E-04 | 8.468E-05 | 2.930E-06 | 2.577E-05 |
| GO:0031974 | membrane-enclosed lumen | 2,497 | 1.258E-11 | 0.000E+00 | 0.000E+00 | 1.387E-03 | 0.000E+00 | 6.321E-05 | 8.296E-12 | 2.560E-08 |
| GO:0031981 | nuclear lumen | 2,066 | 4.504E-11 | 1.110E-16 | 0.000E+00 | 1.419E-03 | 3.209E-14 | 5.008E-03 | 2.809E-09 | 1.590E-05 |
| GO:0043226 | organelle | 9,009 | 0.000E+00 | 0.000E+00 | 0.000E+00 | 2.870E-04 | 0.000E+00 | 6.581E-08 | 0.000E+00 | 1.399E-14 |
| GO:0043227 | membrane-bounded organelle | 8,067 | 0.000E+00 | 0.000E+00 | 0.000E+00 | 3.580E-05 | 0.000E+00 | 6.340E-08 | 0.000E+00 | 3.331E-16 |
| GO:0043229 | intracellular organelle | 8,994 | 0.000E+00 | 0.000E+00 | 0.000E+00 | 2.842E-04 | 0.000E+00 | 9.811E-08 | 0.000E+00 | 2.809E-14 |
| GO:0043231 | intracellular membrane-bounded organelle | 8,059 | 0.000E+00 | 0.000E+00 | 0.000E+00 | 3.655E-05 | 0.000E+00 | 8.765E-08 | 0.000E+00 | 6.661E-16 |
| GO:0043233 | organelle lumen | 2,463 | 5.696E-12 | 0.000E+00 | 0.000E+00 | 8.399E-04 | 0.000E+00 | 2.468E-05 | 1.425E-12 | 1.055E-08 |
| GO:0044422 | organelle part | 5,386 | 1.355E-14 | 0.000E+00 | 0.000E+00 | 5.703E-04 | 1.144E-14 | 3.235E-06 | 0.000E+00 | 2.721E-08 |
| GO:0044424 | intracellular part | 10,614 | 0.000E+00 | 0.000E+00 | 0.000E+00 | 1.252E-06 | 0.000E+00 | 0.000E+00 | 0.000E+00 | 0.000E+00 |
| GO:0044428 | nuclear part | 2,326 | 3.589E-12 | 0.000E+00 | 0.000E+00 | 3.308E-03 | 6.439E-15 | 3.004E-03 | 2.304E-10 | 1.967E-05 |
| GO:0044444 | cytoplasmic part | 5,568 | 0.000E+00 | 0.000E+00 | 0.000E+00 | 1.301E-04 | 3.220E-15 | 0.000E+00 | 0.000E+00 | 0.000E+00 |
| GO:0044446 | intracellular organelle part | 5,317 | 6.539E-14 | 0.000E+00 | 0.000E+00 | 4.602E-04 | 9.992E-15 | 5.789E-06 | 0.000E+00 | 2.595E-08 |
| GO:0044464 | cell part | 14,412 | 5.995E-15 | 1.110E-16 | 0.000E+00 | 3.020E-07 | 2.729E-07 | 1.137E-10 | 0.000E+00 | 1.689E-09 |
| GO:0070013 | intracellular organelle lumen | 2,425 | 4.393E-12 | 0.000E+00 | 0.000E+00 | 6.647E-04 | 0.000E+00 | 6.400E-05 | 6.208E-12 | 3.023E-08 |
