# Supplementary material for: Identifying Highly Conserved and Highly Differentiated Gene Ontology Categories in Human Populations
Source: PLoS One. 2011 Nov 30;6(11):e27871. doi: 10.1371/journal.pone.0027871 (PMC3227580; doi:10.1371/journal.pone.0027871)
Supplement: Table S2 — GO terms associated with low genetic differences among 11 HapMap populations. (DOC) [file pone.0027871.s003.doc]

**Supplementary table 2.** GO terms associated with low genetic differences among 11 HapMap populations.

| GO ID | Term name | Gene  number | Left side p-values | | | | | | | |
| --- | --- | --- | --- | --- | --- | --- | --- | --- | --- | --- |
|  |  |  | Maf | r^2^ | block_size | Snp_dens | hap_div | tag_perc | Cap_perc | max_r^2^ |
| Biological process (7 terms) | |  |  |  |  |  |  |  |  |  |
| GO:0003008 | system process | 1,614 | 1.153E-33 | 4.465E-39 | 7.230E-19 | 9.514E-07 | 3.662E-26 | 5.053E-16 | 2.483E-24 | 1.569E-23 |
| GO:0050877 | neurological system process | 1,268 | 4.913E-39 | 9.332E-39 | 5.462E-18 | 2.676E-07 | 2.289E-26 | 2.218E-20 | 1.219E-28 | 1.134E-25 |
| GO:0007600 | sensory perception | 767 | 3.047E-58 | 1.025E-44 | 1.062E-22 | 4.343E-12 | 3.385E-29 | 2.372E-37 | 1.085E-47 | 3.049E-40 |
| GO:0007606 | sensory perception of chemical stimulus | 439 | 2.859E-115 | 4.693E-81 | 3.932E-30 | 9.529E-21 | 5.449E-48 | 1.152E-86 | 7.515E-99 | 4.218E-89 |
| GO:0007608 | sensory perception of smell | 393 | 2.743E-116 | 1.627E-80 | 1.576E-28 | 2.498E-20 | 7.540E-47 | 3.313E-86 | 8.753E-97 | 5.898E-88 |
| GO:0031018 | endocrine pancreas development | 115 | 6.219E-07 | 9.741E-03 | 7.058E-06 | 9.120E-04 | 4.200E-03 | 8.910E-05 | 2.910E-07 | 6.891E-03 |
| GO:0032501 | multicellular organismal process | 4,789 | 1.052E-21 | 5.136E-36 | 1.121E-12 | 8.561E-06 | 3.649E-23 | 1.070E-08 | 2.754E-15 | 2.618E-15 |
| Molecular function (6 terms) | |  |  |  |  |  |  |  |  |  |
| GO:0004871 | signal transducer activity | 1,891 | 4.629E-26 | 3.672E-33 | 1.623E-23 | 3.850E-06 | 1.456E-18 | 2.076E-14 | 1.359E-26 | 5.909E-19 |
| GO:0004872 | receptor activity | 1,567 | 2.011E-40 | 1.050E-40 | 1.177E-30 | 2.604E-08 | 3.962E-24 | 1.052E-22 | 4.430E-39 | 1.460E-25 |
| GO:0004888 | transmembrane receptor activity | 1,126 | 1.246E-59 | 1.973E-53 | 6.895E-33 | 3.357E-12 | 2.737E-28 | 3.736E-34 | 1.190E-49 | 3.938E-38 |
| GO:0004930 | G-protein coupled receptor activity | 769 | 9.225E-103 | 2.270E-75 | 2.434E-36 | 1.415E-18 | 2.931E-39 | 2.667E-74 | 1.096E-93 | 8.591E-72 |
| GO:0004984 | olfactory receptor activity | 380 | 3.588E-122 | 1.055E-84 | 5.038E-30 | 2.810E-21 | 1.734E-45 | 6.824E-93 | 5.230E-107 | 1.228E-93 |
| GO:0060089 | molecular transducer activity | 1,891 | 4.629E-26 | 3.672E-33 | 1.623E-23 | 3.850E-06 | 1.456E-18 | 2.076E-14 | 1.359E-26 | 5.909E-19 |
| Cellular component (4 terms) | |  |  |  |  |  |  |  |  |  |
| GO:0005882 | intermediate filament | 163 | 1.513E-24 | 1.341E-15 | 1.350E-09 | 8.526E-06 | 1.853E-12 | 3.993E-21 | 7.690E-26 | 3.934E-19 |
| GO:0005615 | extracellular space | 746 | 8.787E-06 | 2.992E-08 | 5.171E-16 | 6.410E-03 | 4.432E-03 | 3.560E-03 | 6.350E-08 | 3.969E-03 |
| GO:0045095 | keratin filament | 84 | 1.015E-16 | 4.849E-12 | 2.216E-06 | 1.017E-04 | 2.126E-08 | 1.298E-16 | 4.632E-19 | 5.269E-15 |
| GO:0045111 | intermediate filament cytoskeleton | 191 | 7.333E-18 | 3.789E-11 | 1.722E-07 | 2.191E-05 | 2.746E-09 | 2.083E-15 | 5.043E-19 | 3.385E-13 |
