# Supplementary material for: Identifying Highly Conserved and Highly Differentiated Gene Ontology Categories in Human Populations
Source: PLoS One. 2011 Nov 30;6(11):e27871. doi: 10.1371/journal.pone.0027871 (PMC3227580; doi:10.1371/journal.pone.0027871)
Supplement: Table S3 — Pearson's correlation coefficients between p-values and gene numbers of GO categories. (DOC) [file pone.0027871.s004.doc]

**Supplementary table 3.** Pearson’s correlation coefficients between p-values and gene numbers of GO categories.

| indicators | maf | r^2^ | block_size | SNP_dens | hap_div | tag_perc | cap_perc | max_ r^2^ |
| --- | --- | --- | --- | --- | --- | --- | --- | --- |
| correlation coefficients | 0.094 | 0.104 | 0.138 | 0.170 | 0.111 | 0.096 | 0.083 | 0.110 |
