# Supplementary material for: Identifying Highly Conserved and Highly Differentiated Gene Ontology Categories in Human Populations
Source: PLoS One. 2011 Nov 30;6(11):e27871. doi: 10.1371/journal.pone.0027871 (PMC3227580; doi:10.1371/journal.pone.0027871)
Supplement: Table S4 — Correlation coefficients matrix for eight indicators. (DOC) [file pone.0027871.s005.doc]

**Supplementary table 4.** Correlation coefficients matrix for eight indicators.

| indicators | Maf | r^2^ | block_size | Snp_dens | hap_div | tag_perc | Cap_perc | max_r^2^ |
| --- | --- | --- | --- | --- | --- | --- | --- | --- |
| Maf | 1.000 | 0.452 | 0.394 | 0.304 | 0.322 | 0.087 | 0.258 | 0.233 |
| r^2^ | 0.452 | 1.000 | 0.446 | 0.249 | 0.527 | 0.135 | 0.327 | 0.606 |
| block_size | 0.394 | 0.446 | 1.000 | 0.473 | 0.179 | 0.070 | 0.524 | 0.060 |
| Snp_dens | 0.304 | 0.249 | 0.473 | 1.000 | 0.199 | 0.173 | 0.279 | 0.162 |
| hap_div | 0.322 | 0.527 | 0.179 | 0.199 | 1.000 | -0.294 | -0.167 | 0.227 |
| tag_perc | 0.087 | 0.135 | 0.070 | 0.173 | -0.294 | 1.000 | 0.739 | 0.569 |
| Cap_perc | 0.258 | 0.327 | 0.524 | 0.279 | -0.167 | 0.739 | 1.000 | 0.400 |
| max_r^2^ | 0.233 | 0.606 | 0.060 | 0.162 | 0.227 | 0.569 | 0.400 | 1.000 |
